# Identification of LEFTY as a molecular marker for ovarian clear cell carcinoma

**DOI:** 10.18632/oncotarget.18882

**Published:** 2017-06-29

**Authors:** Masashi Akiya, Masaaki Yamazaki, Toshihide Matsumoto, Yusuke Kawashima, Yasuko Oguri, Sabine Kajita, Daiki Kijima, Risako Chiba, Ako Yokoi, Hiroyuki Takahashi, Yoshio Kodera, Makoto Saegusa

**Affiliations:** ^1^ Department of Pathology, Kitasato University School of Medicine, Sagamihara, Kanagawa 252-0374, Japan; ^2^ Center for Disease Proteomics, School of Science, Kitasato University, Sagamihara, Kanagawa 252-0374, Japan

**Keywords:** LEFTY, ovarian clear cell carcinoma, cell proliferation, apoptosis, TGF-beta

## Abstract

To identify proteins involved in ovarian clear cell carcinoma (OCCCa), shotgun proteomics analysis was applied using formalin-fixed and paraffin-embedded samples of ovarian carcinoma. Analysis of 1521 proteins revealed that 52 were differentially expressed between four OCCCa and 12 non-OCCCa samples. Of the highly expressed proteins in OCCCa, we focused on left-right determination factor (LEFTY), a novel member of the transforming growth factor-β superfamily. In 143 cases of ovarian epithelial carcinoma including 99 OCCCas and 44 non-OCCCas, LEFTY expression at both mRNA and protein levels was significantly higher in OCCCas compared with non-OCCCas, with the mRNA expression of *LEFTY1* being predominant compared to that of *LEFTY2*. OCCCa cells stably overexpressing LEFTY1 showed reduced cell proliferation, along with decreased pSmad2 expression, and also either displayed an activated p53/p21^waf1^ pathway or increased p27^kip1^ expression, directly or indirectly. Moreover, the treatment of stable cell lines with cisplatin led to increased apoptotic cells, together with the inhibition of protein expression of a pSmad2-mediated X-linked inhibitor of apoptosis and a decreased bcl2/bax ratio. Blocking LEFTY1 expression with a specific short hairpin RNA inhibited cisplatin-induced apoptosis, probably through the increased expression of both XIAP and bcl2, but not bax. In clinical samples, a significantly higher number of apoptotic cells and lower Ki-67 labeling indices were observed in OCCCas with a high LEFTY score relative to those with a low score. These findings suggest that LEFTY may be an excellent OCCCa-specific molecular marker, which has anti-tumor effects in altering cell proliferation and cellular susceptibility to apoptosis.

## INTRODUCTION

Ovarian epithelial carcinoma (OECa) consists of four histologically distinct subtypes, including serous (OSeCa), mucinous (OMuCa), endometrioid (OEmCa), and clear cell (OCCCa), and has the worst prognosis of all gynecological malignancies [1]. OCCCa is considered to be a distinct entity to OECa and has a specific carcinogenic mechanism [2]. Although the most effective treatment for OECa is platinum-based chemotherapy such as cisplatin (CDDP), OCCCa often shows chemoresistance and clinical outcomes at advanced stages are generally unfavorable, despite its slow growth [3–5]. In addition, it is frequently associated with endometriosis, which is thought to be a precursor of tumor lesions [6, 7].

Transforming growth factor-β (TGF-β) is a major regulator of proliferation, survival, migration/invasion and metastasis in cancer cells. TGF-β has diverse roles in human tumorigenesis, including tumor suppressor activity at early stages, and tumor promoter activity at late stages [6–9]. Upon ligand binding, TGF-β receptor recruits and subsequently induces an intracellular downstream signaling cascade involving Smad proteins, which, in turn, specifies nuclear transcriptional targets [10]. To date, eight Smads have been identified and classified into three subfamilies: receptor-regulated Smads (R-Smads: Smad1, 2, 3, 5 and 8); common Smad (Smad4); and inhibitory Smads (Smad6 and 7) [10–12].

The left-right determination factor (LEFTY) is a novel member of the TGF-β superfamily, consisting of *lefty1* and *lefty2* in mice, which are homologous to *LEFTY1* and *LEFTY2* in humans, respectively. *LEFTY1* is identical to *LEFTYB*, whereas *LEFTY2* is identical to *LEFTYA* [13–18]. LEFTY serves as a repressor of TGF-β signaling by inhibiting Smad2 phosphorylation after activation of the TGF-β receptor, and further suppresses downstream events after R-Smad phosphorylation, including the heterodimerization of R-Smads with Smad4, and the nuclear translocation of the R-Smad-Smad4 complex [19].

This study was conducted to clarify the distinguishing factors between OCCCa and other histological subtypes of OECa based on their protein signatures derived from shotgun proteomics. We found significant up-regulation of LEFTY expression at both the mRNA and protein levels in OCCCa tissues when compared with other histological subtypes of OECa. We further elucidated the function and regulation of LEFTY, and examined TGF-β1/Smad signaling and changes in cell kinetics in OCCCa cells.

## RESULTS

### Shotgun proteomics analysis of clinical OECa samples

**Figure 1:**
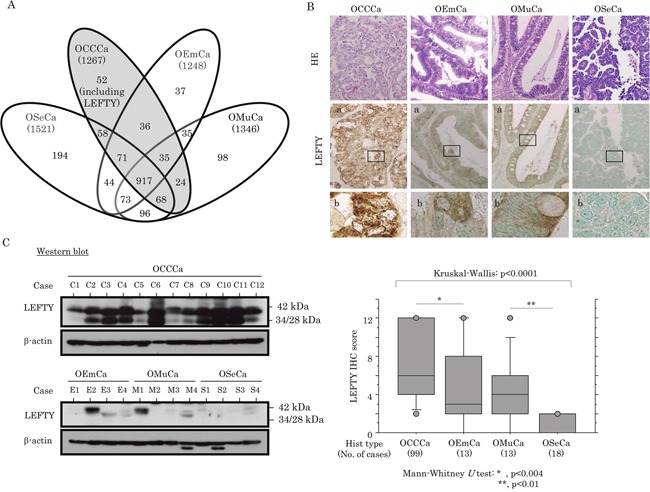
Up-regulation of LEFTY protein expression in OCCCa **(A)** Proteins detected in ovarian epithelial carcinomas (OECas), including ovarian serous carcinoma (OSeCa), clear cell carcinoma (OCCCa), endometrioid carcinoma (OEmCa), and mucinous carcinoma (OMuCa), by shotgun proteomics analysis. **(B)** Upper: staining is by hematoxylin and eosin (HE) and immunohistochemistry (IHC) for LEFTY in OECas. Note the diffuse distribution of strong cytoplasmic LEFTY-positive cells in OCCCa, in contrast to a sporadic or absent distribution in non-OCCCa. Enclosed boxes in panels **(a)** are magnified in panels **(b)**. Original magnification, x100 and x400 (inset). Lower: IHC score for LEFTY in OECas. Hist, histology; No., number **(C)** LEFTY expression detected by western blot assay in OECas. Note the precursor (42 kDa) and the cleaved forms (34 and 28 kDa).

To extract protein for shotgun proteomics analysis, formalin-fixed and paraffin-embedded (FFPE) samples were exposed to a high concentration of Tris-buffer with boiling (Supplementary Figure 1A), which enabled the detection of many proteins with a relatively high molecular weight (Supplementary Figure 1B). This assay was used to identify differences in molecular expression between OCCCa and non-OCCCa. As shown in Figure 1A, a total of 5382 proteins, including 1267 in OCCCa, 1248 in OEmCa, 1346 in OMuCa and 1521 in OSeCa, were detected from 16 OECa tissues consisting of four samples of each subtype. Of these, 52 proteins were observed in all four OCCCa samples, but in less than three non-OCCCa samples (Table 1). Based on the values of spectral counts detected in the assay, we focused on LEFTY1 and LEFTY2, a novel member of the TGF-β superfamily, because LEFTY is a well-characterized molecule that plays an important role in the control of cell differentiation and proliferation during embryonic development [20, 21]. However, the significance of its expression in tumor tissues remains to be clarified.

**Table 1:** Summary of the protein profiles with high expression levels detected by shotgun proteomic assays in ovarian carcinomas

Accession number	Protein	Gene symbol	OCCCa					OEmCa					OSeCa					OMuCa				
			Spectral counts				Positive	Spectral counts				Positive	Spectral counts				Positive	Spectral counts				Positive
			C1	C2	C3	C4	cases	E1	E2	E3	E4	cases	S1	S2	S3	S4	cases	M1	M2	M3	M4	cases
P84085	ADP-ribosylation factor 5	ARF5	3	3	3	2	4	1	1	1	2	1	3	1	1	2	2	2	1	2	1	2
P19801	Amiloride-sensitive amine oxidase [copper-containing]	ABP1	11	18	15	15	4	0	0	0	0	0	0	0	0	0	0	0	7	5	15	3
P06727	Apolipoprotein A-IV	APOA4	2	2	10	3	4	13	0	0	4	2	2	5	0	3	3	3	0	1	11	2
E9PK09	Bcl-2-associated transcription factor 1 (Fragment)	BCLAF1	3	5	3	4	4	1	4	2	2	3	0	2	4	2	3	2	1	1	1	1
P15291	Beta-1, 4-galactosyl transferase 1	B4GALT1	4	2	4	4	4	1	2	1	1	1	0	0	1	1	0	1	4	1	1	1
Q9P2M7	Cingulin	CGN	4	7	2	2	4	1	2	2	2	3	2	2	3	2	4	0	5	3	2	3
Q01955	Collagen alpha-3(IV) chain	COL4A3	2	2	3	4	4	2	2	2	1	3	2	2	1	2	3	1	1	2	2	2
F6RFD5	Destrin	DSTN	2	4	2	2	4	1	1	3	2	2	3	3	1	3	3	1	2	2	3	3
P53634	Dipeptidyl peptidase 1	CTSC	4	2	6	3	4	1	2	1	0	1	2	2	3	1	3	3	2	1	2	3
Q9H6S3	Epidermal growth factor receptor kinase substrate 8-like protein	EPS8L2	3	6	2	4	4	1	0	0	2	1	2	1	2	0	2	1	5	7	10	3
B1AHL2	Fibulin 1	FBLN1	5	11	19	16	4	1	18	4	9	3	8	1	7	5	3	3	1	1	1	1
F5H4×1	General vesicular transport factor p115	USO1	2	4	2	5	4	1	4	3	2	3	4	3	3	1	3	1	3	5	0	2
P35754	Glutaredoxin-1	GLRX	4	4	4	3	4	2	1	1	1	1	2	2	1	0	2	2	1	2	2	3
E9PB90	Hexokinase-2	HK2	2	2	2	4	4	2	1	1	1	1	1	2	3	3	3	2	2	1	4	3
Q16270	Insulin-like growth factor-binding protein 7	IGFBP7	5	8	3	4	4	1	1	1	1	0	2		1	1	1	1	0	0	1	0
P06756	Integrin alpha-V	ITGAV	4	6	6	4	4	1	0	3	2	2	9	1	4	3	3	2	1	1	0	1
P19827	Inter-alpha-trypsin inhibitor heavy chain H1	ITIH1	4	4	2	2	4	4	1	1	1	1	6	2	3	1	3	1	0	3	3	2
Q06033	Inter-alpha-trypsin inhibitor heavy chain H3	ITIH3	7	2	2	3	4	0	0	2	0	1	13	1	1	1	1	0	1	1	2	1
Q63ZY3	KN motif and ankyrin repeat domain-containing protein 2	KANK2	2	4	3	4	4	1	5	5	6	3	7	3	1	6	3	1	1	2	2	2
Q13751	Laminin subunit beta-3	LAMB3	3	5	3	5	4	2	0	1	1	1	0	0	0	1	0	1	4	1	0	1
**O75610**	**Left-right determination factor 1**	**LEFTY1**	**28**	**58**	**8**	**36**	**4**	**0**	**0**	**0**	**0**	**0**	**0**	**0**	**0**	**0**	**0**	**2**	**0**	**0**	**0**	**1**
**O00292**	**Left-right determination factor 2**	**LEFTY2**	**18**	**49**	**6**	**29**	**4**	**0**	**0**	**0**	**0**	**0**	**0**	**0**	**0**	**0**	**0**	**1**	**0**	**0**	**0**	**0**
P18428	Lipopolysaccharide-binding protein	LBP	4	6	2	4	4	2	1	0	1	1	2	2	1	0	2	0	0	2	5	2
K7EJE8	Lon protease homolog	LONP1	10	11	2	3	4	9	1	1	1	1	0	7	5	0	2	0	4	2	2	3
P10253	Lysosomal alpha-glucosidase	GAA	6	6	2	3	4	1	3	3	2	3	2	4	0	2	3	2	3	0	8	3
Q9HCC0	Methylcrotonoyl-CoA carboxylase beta chain, mitochondrial	MCCC2	3	4	4	3	4	2	1	1	1	1	0	2	1	2	2	1	3	1	1	1
P22570	NADPH:adrenodoxin oxidoreductase, mitochondrial	FDXR	9	5	8	4	4	2	0	1	3	2	1	1	0	3	1	5	7	1	1	2
H0Y6T7	Nicastrin (Fragment)	NCSTN	3	2	3	2	4	2	2	1	2	3	2	2	2	1	3	2	2	2	1	3
Q6XQN6	Nicotinate phosphoribosyl transferase	NAPRT1	3	3	3	4	4	3	0	1	0	1	1	3	4	2	3	0	10	4	4	3
Q9Y617	Phosphoserine aminotransferase	PSAT1	8	16	10	10	4	0	1	0	2	1	0	5	3	8	3	1	0	0	0	0
Q9Y446	Plakophilin-3	PKP3	8	10	2	2	4	2	0	0	0	1	0	9	0	1	1	0	4	8	10	3
Q9UHX1	Poly(U)-binding-splicing factor PUF60	PUF60	3	4	2	3	4	0	3	1	0	1	1	8	4	6	3	2	3	2	0	3
Q8NBJ5	Procollagen galactosyltransferase 1	GLT25D1	4	8	5	4	4	2	3	1	2	3	3	0	2	3	3	4	4	1	1	2
Q02809	Procollagen-lysine, 2-oxoglutarate 5-dioxygenase 1	PLOD1	11	15	3	4	4	0	1	1	3	1	0	0	6	3	2	0	4	1	1	1
P25788	Proteasome subunit alpha type-3	PSMA3	4	2	2	2	4	2	4	1	1	2	1	4	2	2	3	2	1	7	4	3
Q9P258	Protein RCC2	RCC2	5	5	3	4	4	1	5	3	6	3	1	1	9	7	2	4	9	4	1	3
B4DJA5	Ras-related protein Rab-5A	RAB5A	4	4	2	2	4	2	4	1	2	3	1	3	1	2	2	1	0	2	2	2
P61020	Ras-related protein Rab-5B	RAB5B	7	4	2	3	4	2	4	1	2	3	2	5	1	3	3	1	1	4	2	2
Q9NQG5	Regulation of nuclear pre-mRNA domain-containing protein 1B	RPRD1B	5	3	2	2	4	2	2	1	1	2	1	4	4	5	3	2	6	2	1	3
D6REQ6	Ribonuclease T2	RNASET2	5	8	2	2	4	1	2	1	3	2	0	0	0	0	0	3	4	1	3	3
Q9Y230	RuvB-like 2	RUVBL2	3	4	2	2	4	0	2	2	0	2	1	2	2	4	3	3	0	2	1	2
O00391	Sulfhydryl oxidase 1	QSOX1	2	11	3	8	4	0	2	0	0	1	0	0	0	0	0	0	11	14	1	2
Q5T8U5	Surfeit 4	SURF4	3	2	2	2	4	2	2	1	1	2	1	1	2	2	2	1	1	1	2	1
F6SKP1	Transmembrane glycoprotein NMB	GPNMB	3	2	6	2	4	3	2	0	0	2	1	1	1	0	0	1	0	0	0	0
Q504Y1	TRPM3 protein	TRPM3	6	4	2	5	4	5	4	5	1	3	4	6	0	4	3	2	0	0	2	2
B1AH89	Tubulin tyrosine ligase-like family, member 12	TTLL12	3	8	2	4	4	2	3	0	3	3	1	7	2	3	3	2	4	4	1	3
Q9Y224	UPF0568 protein C14orf166	C14orf166	4	4	2	2	4	1	0	1	3	1	1	3	4	4	3	1	5	4	2	3
D6RGZ6	Versican core protein (Fragment)	VCAN	3	6	6	8	4	1	1	3	3	2	18	0	1	1	1	0	0	0	0	0
Q6PCB0	von Willebrand factor A domain-containing protein 1	VWA1	5	2	2	5	4	1	1	2	3	2	3	0	1	2	2	2	0	0	1	1
P04275	von Willebrand factor	VWF	4	2	5	5	4	0	2	4	1	2	7	1	1	3	2	1	0	2	2	2
P36543	V-type proton ATPase subunit E 1	ATP6V1E1	2	3	3	2	4	1	2	3	2	3	1	4	1	2	2	2	2	0	2	3
O75348	V-type proton ATPase subunit G 1	ATP6V1G1	2	2	2	2	4	0	0	1	0	0	1	2	2	2	3	0	2	0	2	2

OCCCa, ovarian clear cell carcinoma; OEmCa, ovarian endometrioid carcinoma; OSeCa, ovarian serous carcinoma; OMuCa, ovarian mucinous carcinoma.

### Up-regulation of LEFTY in OCCCa

Representative images of immunohistochemistry (IHC) findings for LEFTY in the four histological types of OECa are illustrated in Figure 1B (upper panels). The anti-LEFTY antibody used in this study was able to react with both LEFTY isoforms (Supplementary Figure 1C). This is because LEFTY1 and LEFTY2 are both 366 amino acids long, and differ at only 16 residues, thus they are 97% identical and share 350 identical residues.[18] Cytoplasmic LEFTY immunoreactivity, with or without nuclear staining, was frequently observed in OCCCa, in contrast to its focal positivity or absence in non-OCCCas. Average IHC scores for LEFTY showed a significant incremental decrease from OCCCa, OEmCa, OMuCa to OSeCa (Figure 1B, lower graph). Similar findings were also evident by western blot assay, which demonstrated bands at 42, 34, and 28 kDa corresponding to the precursor peptide and polypeptides cleaved at Arg^77^ and Arg^135^, respectively (Figure 1C and Supplementary Figure 1C) [18].

In 85 OCCCa cases that were investigated, LEFTY scores were not associated with several clinicopathological factors looked at, including overall survival, progression-free survival, clinical stage, and lymph node metastasis (Supplementary Figure 2A and 2B). Of these, six cases of postoperatively recurred tumors were available for further examination (Supplementary Table 1). The LEFTY score appeared to be decreased in the recurred OCCCas as compared to primary tumors, but the difference did not reach significance (*p*=0.37), probably due to the small number of cases investigated. In contrast, changes in apoptosis and cell proliferation status varied between the two lesions (Supplementary Figure 2C and 2D, and Supplementary Table 1).

**Figure 2:**
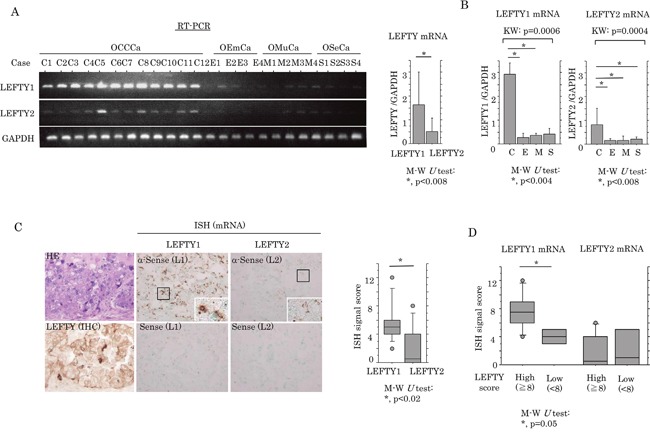
Up-regulation of LEFTY mRNA expression in OCCCa **(A)** Left: expression of *LEFTY1* and *LEFTY2* mRNA in OECa by RT-PCR assay. Note the frequent mRNA expression of *LEFTY1*, but not *LEFTY2*, in OCCCa, in contrast to its low or absent expression level in non-OCCCa. OCCCa, clear cell carcinoma; OEmCa, endometrioid carcinoma; OMuCa, mucinous carcinoma; OSeCa, serous carcinoma. Right: relative LEFTY1 and LEFTY2 mRNA levels in OECas were calculated by normalization to GAPDH using an NIH ImageJ program. Data was expressed as mean±SD. **(B)** Relative mRNA levels of LEFTY1 (left) and LEFTY2 (right) in OECas were calculated by normalization to GAPDH using an NIH ImageJ program. C, OCCCa; E, OEmCa; M, OMuCa; S, OSeCa; KW, Kruskal-Wallis; M-W, Mann-Whitney *U*-test. Data was expressed as mean±SD. **(C)** Left: staining by HE, IHC for LEFTY, and *in situ* hybridization (ISH) for mRNA of LEFTY1 (L1) and LEFTY2 (L2) in OCCCa. Enclosed boxes are magnified. Original magnification, x200 and x400 (inset). Right: ISH signal score for LEFTY1 and LEFTY2 in OCCCa. M-W, Mann-Whitney *U*-test. **(D)** Relationship of ISH signals for LEFTY1 and LEFTY2 mRNAs with an IHC score for LEFTY. M-W, Mann-Whitney *U*-test.

Next, the association between mRNA expression of LEFTY and OECa was examined using specific primers for *LEFTY1* and *LEFTY2* (Supplementary Figure 1D). The expression level of LEFTY1 mRNA was significantly higher than that of LEFTY2 in OECas (Figure 2A). The expression of both LEFTY1 and LEFTY2 mRNAs was significantly higher in OCCCa compared with non-OCCCa (Figure 2B). In the 10 OCCCa cases that were investigated, positive mRNA signals for LEFTY1, as detected by *in situ* hybridization, were also significantly higher than for LEFTY2 (Figure 2C and Supplementary Table 2), in line with the shotgun proteomics data showing that 11 and 2 specific peptide fragments for LEFTY1 and LEFTY2, respectively, as well as 16 identical fragments for both isoforms, were detected in 4 OCCCa tissues (Supplementary Table 3). These were associated with LEFTY immunointensity, when cases were divided into high (score≧8) and low (score< 8) categories according to a mean value (8.6; Figure 2D).

**Figure 3:**
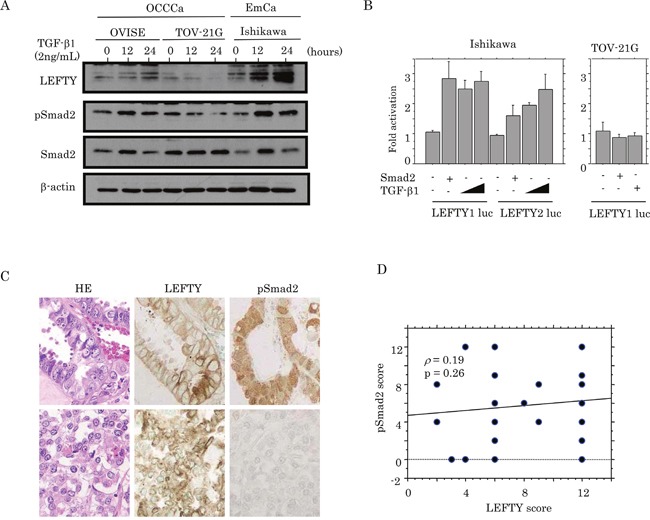
Relationship between LEFTY expression and TGF-β1/Smad signaling **(A)** Western blot analysis of LEFTY, pSmad2, and Smad2 in OVISE, TOV-21G, and Ishikawa cells treated with 2 ng/mL TGF-β1 for 0, 12, and 24 hours. **(B)** Ishikawa and TOV-21G cells were transfected with LEFTY1 and LEFTY2 reporter constructs, together with Smad2 and TGF-β1 treatment. Relative activity was determined based on arbitrary light units of luciferase activity normalized to pRL-TK activity. The activities of the reporter plus the effector relative to that of the reporter plus empty vector are shown as mean±SD. The experiment was performed in duplicate. **(C)** HE staining and IHC for LEFTY and pSmad2 in semi-serial sections of OCCCa. Note the pSmad2 immunoreactivity in both nuclear and cytoplasmic compartments. Original magnification, x200. **(D)** Correlation between LEFTY and pSmad2 scores in OCCCa.

To examine a link between LEFTY expression and the TGF-β1/Smad pathway, OCCCa cells including OVISE and TOV-21G cell lines were treated with TGF-β1. Ishikawa cells, an endometrial cancer cell line, were also used as a positive control, since treatment of the cells with TGF-β1 had previously resulted in the apparent increased expression of LEFTY and pSmad2, along with the induction of the p21^waf1^ and p27^kip1^ expression (Supplementary Figure 3). As shown in Figure 3A, increased expression of both pSmad2 and LEFTY in response to TGF-β1 was observed in OVISE and Ishikawa cells, whereas such effects were only minor in TOV-21G cells. Smad2 transfection or TGF-β1 treatment also led to markedly increased promoter activity of both *LEFTY1* and *LEFTY2* in Ishikawa, but not TOV-21G cells (Figure 3B). In clinical samples, although LEFTY immunopositivity appeared to colocalize with pSmad2 immunoreactivity within tumor tissues of several OCCCa cases (Figure 3C), the LEFTY score did not correlate with the pSmad2 score (Figure 3D).

Because active demethylation is essential for regulating a subset of TGF-β1-dependent genes, [22] we further investigated changes in CpG island status within *LEFTY1* in response to TGF-β1 by sodium bisulfate sequencing (Supplementary Figure 4A). Although *LEFTY1* contained a region of CpG islands that were highly methylated in OVISE, TOV-21G and Ishikawa cells, demethylation by TGF-β1 treatment was rare (Supplementary Figure 4B).

### Relationship of LEFTY expression with cell proliferation in OCCCa

**Figure 4:**
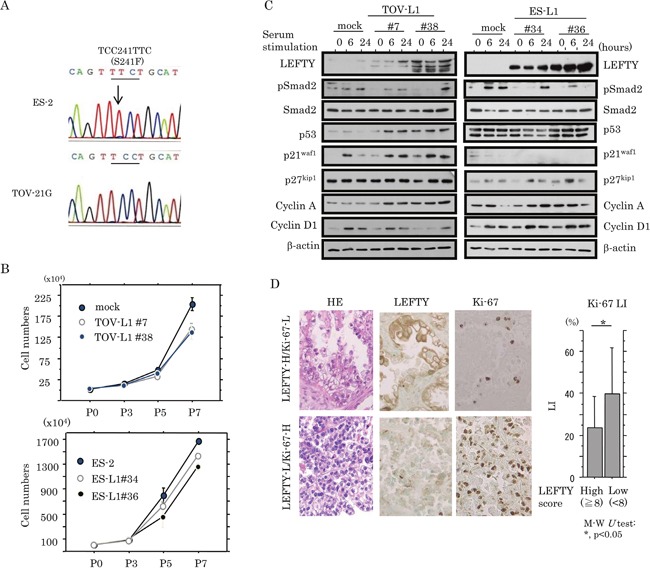
Association between overexpression of LEFTY and cell proliferation in OCCCa **(A)** Sequence analysis of *p53*. Note the heterozygous substitution mutation (indicated by an arrow) in ES-2, but not TOV-21G, cells. **(B)** Two independent TOV-21G (TOV-L1#7 and #38) and ES-2 (ES-L1#34 and #36) cell lines stably overexpressing LEFTY1 and their mock cells were seeded at low density. The cell numbers are presented as mean±SD. P0, P3, P5, and P7 indicate 0, 3, 5, and 7 days after cell passage, respectively. **(C)** Western blot analysis for the indicated proteins in both TOV-L1 and ES-L1 stable cells and their mock cells for the times shown following restimulation with 10% serum after serum starvation for 6 h. **(D)** Left: HE staining and IHC for LEFTY, and Ki-67 in OCCCa. Sporadic Ki-67-immunopositive cells are shown in OCCCa with high LEFTY immunoreactivity, in contrast to the diffuse Ki-67 staining in tumors with low LEFTY staining. Original magnification, x200. Right: Ki-67 labeling indices (LIs) between OCCCas with high and low LEFTY scores. Data are expressed as the mean±SD. M-W, Mann-Whitney *U*-test.

To examine whether LEFTY expression affects cell proliferation in OCCCa cells, two independent cell lines stably overexpressing LEFTY1 were established using TOV-21G cells with low endogenous LEFTY expression as well as wild-type (wt) *p53*, and ES-2 cells with a loss of LEFTY expression and mutant-type (mt) *p53* (TOV-L1 and ES-L1, respectively; Figure 4A). Both TOV-L1 and ES-L1 stable cells showed a tendency towards a low proliferation rate, particularly in the exponential growth phase (Figure 4B and Supplementary Figure 5A). To further examine alterations in expression of several cell cycle-related molecules during cell growth, stable cells were rendered quiescent by serum starvation and were subsequently stimulated with serum. At 6 and 24 h after release in the cell cycle, expression levels of p53 and p21^waf1^ in TOV-L1 stable cells and p27^kip1^ in ES-L1 stable cells were substantially increased relative to the mock cells, in contrast to the decreased expression of pSmad2 in both stable cells (Figure 4C). In clinical samples, average Ki-67 labeling indices (LIs) were significantly lower in OCCCa with high LEFTY scores (≧8) relative to tumors with low scores (<8; Figure 4D).

**Figure 5:**
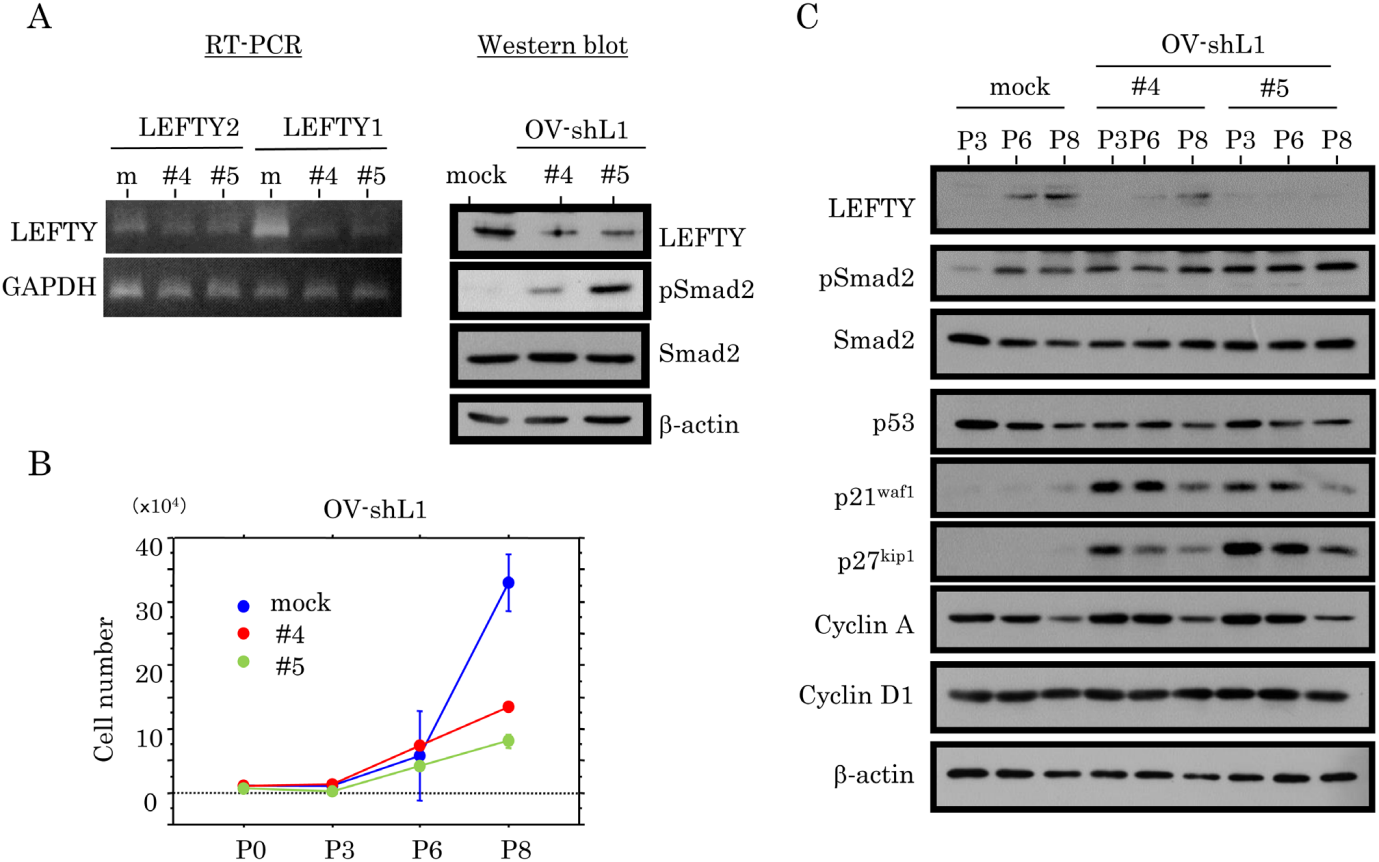
Association between knockdown of LEFTY expression and cell proliferation in OCCCa **(A)** Decreased expression of endogenous LEFTY at both mRNA (left, RT-PCR) and protein (right, western blot) levels in two independent OVISE cell lines with knockdown of endogenous LEFTY1 expression (OV-shL1#4 and #5). **(B)** Two independent OV-shL1#4 and #5 cells and the mock cells were seeded at low density. The cell numbers are presented as mean±SD. P0, P3, P6, and P8 indicate 0, 3, 6, and 8 days after cell passage, respectively. **(C)** Western blot analysis for the indicated proteins in OV-shL1 and mock cells for the times shown.

Two independent cell lines, with LEFTY expression blocked by a LEFTY1-specific short hairpin RNA (shRNA), were established using OVISE cells (OV-shL1), which have relatively high LEFTY expression and wt *p53*. The two-independent OV-shL1 cell lines (#4 and #5) showed reduced LEFTY at both mRNA and protein levels, and increased pSmad2 expression (Figure 5A). The cell lines also showed a tendency towards a low proliferation rate (Figure 5B and Supplementary Figure 5A), along with an increase in both p21^waf1^ and p27^kip1^ expression, independent of p53 status (Figure 5C).

### Relationship of LEFTY expression with susceptibility to apoptosis in OCCCa

**Figure 6:**
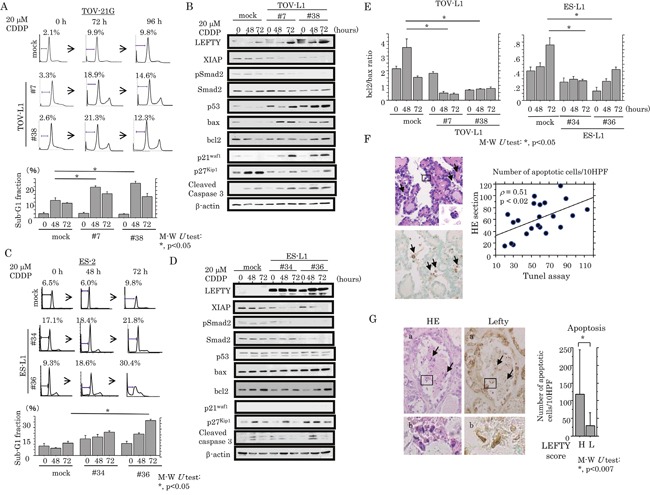
Association between overexpression of LEFTY and susceptibility to apoptosis in OCCCa **(A)** After treatment of TOV-L1 and the mock cells with 20 μM CDDP for the times indicated, cells undergoing apoptosis (sub-G1) were detected by flow cytometry (upper) and as the percentage sub-G1 fraction (lower). This experiment was performed in triplicate using independent samples. **(B)** Western blot analysis of the indicated proteins from TOV-L1 stable cells and the mock cells after 20 μM CDDP treatment for the times shown. **(C)** After treatment of ES-L1 stable cells and the mock cells with 20 μM CDDP for the times indicated, cells undergoing apoptosis (sub-G1) were detected by flow cytometry (upper) and the percentage sub-G1 fraction following CDDP treatment (lower). This experiment was performed in triplicate using independent samples. **(D)** Western blot analysis for the indicated proteins from ES-L1 stable cells and the mock cells after 20 μM CDDP treatment for the times shown. **(E)** Values of endogenous bcl2 relative to bax protein were calculated by normalization to β-actin in TOV-L1 (left) and ES-L1 (right) stable cells after 20 μM CDDP treatment for the times shown. M-W, Mann-Whitney *U*-test. **(F)** Left: detection of apoptotic cells (boxes enclose magnified apoptotic cells) by HE staining (upper) and TUNEL assay (lower) in OCCCa. Note the TUNEL-positive apoptotic cells (indicated by arrows). Original magnification, x100 and x400 (inset). Right: correlation of the detection of apoptotic cells between HE sections and TUNEL assay. **(G)** Left: staining is by HE and IHC for LEFTY in OCCCa. Of note, some apoptotic cells have LEFTY immunoreactivity (indicated by arrows). Enclosed boxes in panels **(a)** are magnified in panels **(b)**. Original magnification, x100 and x400 (inset). Right: apoptotic cells detected by HE sections in OCCCa with high and low LEFTY scores. M-W, Mann-Whitney *U*-test.

We examined the association between LEFTY expression and susceptibility to apoptosis in response to cytotoxic effects. Treatment of TOV-L1 stable cells with CDDP resulted in an increased proportion of both sub-G1 (apoptotic cells) and G2/M fractions, and a decrease in the G1 fraction (Figure 6A and Supplementary Figure 6A), in line with results of decreased cell viability (Supplementary Figure 5B). The expression levels of an X-linked inhibitor of apoptosis protein (XIAP), pSmad2 and p27^kip1^ were apparently decreased in the stable cells as compared to mock cells, in contrast to the increased expression of p53, bax, p21^waf1^, and cleaved caspase 3 (Figure 6B). Similar findings were also evident in ES-L1 stable cells, with the exception of changes in the expression of several cell cycle-related molecules (Figure 6C and 6D, and Supplementary Figure 5B and 6B). Bcl2/bax ratios were also significantly decreased by CDDP treatment in both stable cells (Figure 6E). In clinical samples, apoptotic cells were readily detected in hematoxylin and eosin (HE)-stained sections according to their characteristic features (Figure 6F, left: arrows); the values positively correlated with apoptotic LIs as detected by TdT-mediated dUTP-biotin nick end labeling (TUNEL) assay (Figure 6F, right). As shown in Figure 6G, several apoptotic cells also showed positive immunoreactivity for LEFTY in OCCCa tissues. The average number of apoptotic cells was significantly higher in tumors with high LEFTY scores (≧8) as compared with those in a low LEFTY score (<8) category.

**Figure 7:**
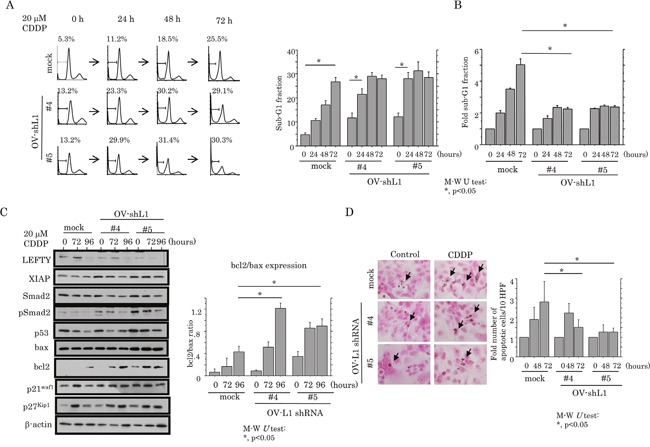
Association between knockdown of LEFTY expression and susceptibility to apoptosis in OCCCa **(A)** After treatment of OV-shL1 cells and the mock cells with 20 μM CDDP for the times shown, cells undergoing apoptosis (sub-G1) were detected by flow cytometry (left) and the percentage sub-G1 fraction (right). This experiment was performed in triplicate using independent samples. M-W, Mann-Whitney *U*-test. **(B)** The relative sub-G1 fraction following 20 μM CDDP for the times shown. The sub-G1 fraction in the absence of CDDP treatment (0 h) was set as 1. M-W, Mann-Whitney *U*-test. **(C)** Left: western blot analysis for the indicated proteins from OV-shL1 cells and the mock cells after 20 μM CDDP treatment for the times shown. Right: values of endogenous bcl2 relative to bax protein were calculated by normalization to β-actin in OV-shL1 cells after 20 μM CDDP treatment for the times shown. M-W, Mann-Whitney *U*-test. **(D)** Left: after treatment with 20 μM CDDP, OV-shL1 cells and the mock cells undergoing apoptosis (indicated by arrows) were detected by TUNEL assay. Original magnification, x400. Right: number of apoptotic cells per 10 high power fields (HPFs) detected by TUNEL assay for the times shown. The number of apoptotic cells in the absence of CDDP treatment (0 h) was set as 1. M-W, Mann-Whitney *U*-test.

Although, in the absence of CDDP treatment, apoptotic cell numbers were increased in OV-shL1 cells compared with mock cells (Figure 7A), the induction of apoptosis, as well as an increase in the G1 cell cycle fraction, in response to CDDP treatment was apparently decreased in the former (Figure 7A and 7B, and Supplementary Figure 6C); increased expression of XIAP, pSmad2 and bcl2 (increased ratio of bcl2/bax; Figure 7C) was also observed. In contrast, changes in cell viability did not differ between OV-shL1 and mock cells (Supplementary Figure 5B). Similar associations were also observed by TUNEL assay (Figure 7D).

### Transcriptional inhibition of Smad2-dependent XIAP expression by LEFTY

**Figure 8:**
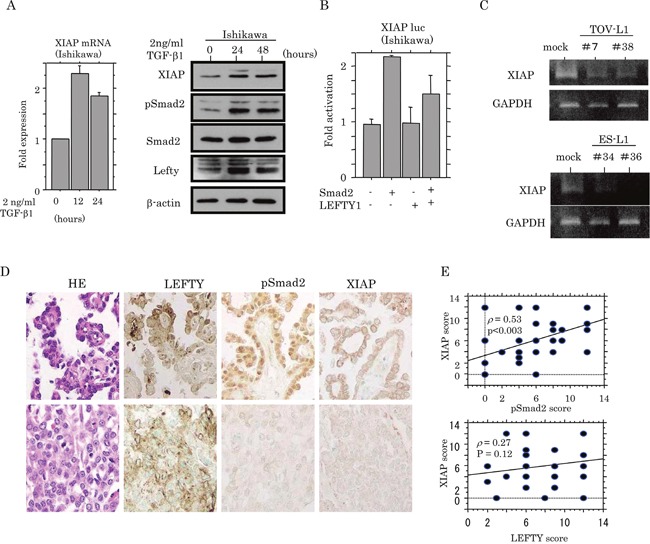
Transcriptional up-regulation of XIAP by TGF-β/Smad signaling **(A)** Real-time RT-PCR analysis (left) of XIAP mRNA expression, and western blot analysis (right) of indicated proteins for the times shown after 2 ng/mL TGF-β treatment of Ishikawa cell lines. **(B)** Ishikawa cells were transfected with XIAP reporter constructs, together with Smad2 and LEFTY1. Relative activity was determined by arbitrary light units of luciferase activity normalized to pRL-TK activity. The activities of the reporter plus the effector relative to that of the reporter plus empty vector are shown as mean±SD. The experiment was performed in duplicate. **(C)** Conventional RT-PCR analysis of endogenous XIAP mRNA expression in TOV-L1 and ES-L1 stable cells. **(D)** Staining was by HE and IHC for LEFTY, pSmad2, and XIAP in semi-serial sections of OCCCa. Original magnification, x200. **(E)** Correlations of XIAP score with pSmad2 (upper) and LEFTY scores (lower) in OCCCa.

Based on the findings related to apoptotic status, we examined whether the expression of XIAP, a well-studied member of the inhibitor of apoptosis protein family, [23, 24] was affected by Smad2. Treatment of Ishikawa cells with TGF-β1 led to an increase in XIAP expression at both mRNA and protein levels, accompanied by the increased expression of pSmad2 and LEFTY (Figure 8A). Activation of the *XIAP* promoter was increased in a two-fold manner by Smad2 transfection, but this effect was apparently abrogated by the cotransfection of LEFTY1 (Figure 8B), concordant with data demonstrating a considerable decrease in endogenous XIAP mRNA expression in both TOV-L1 and ES-L1 stable cells (Figure 8C). In OCCCa tissues, cytoplasmic XIAP immunopositive cells displayed a heterogeneous distribution within tumor lesions and appeared to colocalize with pSmad2, but not LEFTY, immunoreactivity (Figure 8D). This was in line with the significant positive correlation of the XIAP score with the pSmad2, but not LEFTY, score in OCCCa (Figure 8E).

## DISCUSSION

In the present study, using shotgun proteomics, many peptide fragments of both LEFTY1 and LEFTY2 proteins were more frequently detected in surgically-resected OCCCa tissues compared with non-OCCCa tissues. This result was supported by IHC and western blot assays, which showed high LEFTY expression in OCCCa, regardless of several clinicopathological factors, including the survival. Moreover, the mRNA expression level of LEFTY1 was significantly higher than that of LEFTY2 in OCCCa, in line with our shotgun proteomics data showing that specific peptide fragments for LEFTY1 were more frequently detected as compared to those of LEFTY2. A similar finding was also observed in OCCCa as determined from proteomics data [25].

We found TGF-β1-induced transcriptional up-regulation of *LEFTY* in OVISE and Ishikawa cells, probably through increased pSmad2 expression, whereas this association was lacking in TOV-21G cells. This may be due to the existence of anti-Smad proteins (Smad6 and Smad7) and repressors (SnoN and Ski) for TGF-β1-responsive genes in TOV-21G cell lines [10–12, 26]. Given our data showing the absence of a direct correlation between LEFTY and pSmad2 expression in OCCCa tissues, it appeared that other factors may also contribute to the induction of LEFTY expression.

Epigenetic factors are considered to be a mechanism underlying the regulation of LEFTY expression mediated by TGF-β1. For example, TGF-β1 reduced *LEFTY1* CpG island shore methylation by about 10 to 20%, resulting in increased LEFTY expression in human pancreatic and hepatic carcinoma cells [27]. However, we found that a reduction of *LEFTY1* gene methylation in response to TGF-β1 did not occur in both OCCCa and endometrial carcinoma cells, which normally respond well to TGF-β1 stimulation, suggesting that epigenetic changes may be less important in the regulation of LEFTY1 expression in these cells.

Importantly, the overexpression of LEFTY appeared to contribute to the inhibition of aggressive growth in OCCCa, in line with a report showing that blocking TGF-β1 action by LEFTY overexpression may hinder aggressive tumor growth or lead to tumor regression.[19] This conclusion is supported by our observations of significantly decreased Ki-67 LI values in OCCCa with high LEFTY expression, as well as decreased LEFTY expression in postoperatively recurrent tumors. In addition, both TOV-L1 and ES-L1 stable cells with low pSmad2 expression showed a reduced cell proliferation rate, probably due to either an activated p53/p21^waf1^ pathway or the direct or indirect induction of p27^kip1^ expression.

In contrast, OV-shL1 cells also resulted in decreased cell proliferation, along with increased expression of pSmad2, p21^waf1^, and p27^kip1^. Given evidence showing that TGF-β family members can inhibit proliferation in a cell type-dependent manner through multiple mechanisms, including via the upregulation of p15^INK4b^, p21^waf1^, and p27^kip1^, [28] it is possible that pSmad2-dependent p21^waf1^ and p27^kip1^ expression may also contribute to the inhibition of cell proliferation. In fact, changes in the expression of p21^waf1^ and p27^kip1^ in response to the TGF-β1/Smad2 axis were also evident in Ishikawa cells.

In line with our data showing the significantly higher number of apoptotic cells in OCCCa tissues with high LEFTY expression, the overexpression of LEFTY was closely linked with a susceptibility to apoptosis due to CDDP treatment in both TOV-L1 and ES-L1 stable cells. The apoptotic process was accompanied by a decrease in expressions of pSmad2 and XIAP and in the bcl2/bax ratio, and in an increase in cleaved caspase 3 expression. Given that the decrease in the bcl2/bax ratio results in the release of cytochrome *c*, activation of caspase 3, and subsequent apoptosis, [29] it is possible that an alteration in the Smad/XIAP axis by overexpressing LEFTY may be due to disruption of the mitochondrial membrane potential through changes in expression of bax and bcl2 expression.

Unexpectedly, although OV-shL1 cells in the absence of CDDP treatment also increased the numbers of apoptotic cells observed, this may be explained by the upregulation of pSmad2 due to the silencing of LEFTY1 since TGF-β family members are able to induce apoptosis [28]. In contrast, cells showed a lowered susceptibility to apoptosis in response to CDDP treatment, most likely through the increased expression of XIAP and bcl2, but not bax. In fact, XIAP protects endometrial cancer cells against various proapoptotic agents, including chemotherapeutic drugs [30]. Collectively, a complex regulatory mechanism in cell kinetics, including apoptosis and cell proliferation, may exist in OCCCa cells, which acts through signal loops involving the TGF-β/Smad/LEFTY axis, as well as XIAP and bcl family members.

Several lines of evidence from the present study support the conclusion that XIAP expression is under the transcriptional control of pSmad2. Treatment of cells with TGF-β1 led to an increase in XIAP expression at both mRNA and protein levels, along with increased pSmad2 expression. Furthermore, transient transfection of Smad2 led to the transactivation of the *XIAP* promoter, despite the lack of consensus among Smad binding sequences in the *XIAP* promoter. This may be in accordance with data from a previous study showing that constitutive XIAP expression was indirectly regulated by the TGF-β1/Smad/NF-κB pathway.[31] Thirdly, XIAP immunoreactivity was positively correlated with pSmad2 status in OCCCa tissues. Finally, both TOV-L1 and ES-L1 stable cells exhibited a decrease in endogenous XIAP mRNA expression, corresponding to results showing that co-transfection of LEFTY1 abrogated Smad2-mediated transactivation of the *XIAP* promoter. In contrast, OV-shL1 cells resulted in the increased expression of both XIAP and pSmad2. Given that XIAP acts as an intracellular anti-apoptotic protein that functions as a direct inhibitor of caspases-3, -7 and -9, [32] it is therefore suggested that forced expression of LEFTY1 facilitates the initiation of apoptosis by caspase 3, altering the Smad2/XIAP pathway. Upregulation of XIAP expression due to a loss of LEFTY1 causes a resistance to chemotherapy by tumor cells, resulting in tumor progression and recurrence. Although TGF-β1/Smad signaling was shown to be closely correlated with apoptosis through the down-regulation of XIAP levels, [33] this discrepancy may result from cell type specificities.

**Figure 9:**
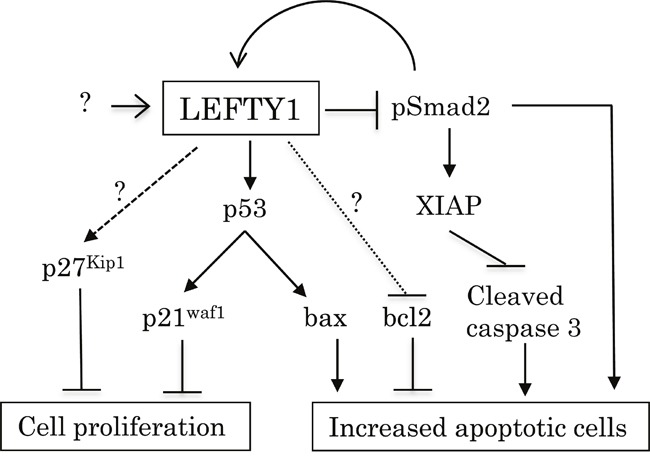
Schematic representation of association of LEFTY expression with cell proliferation and susceptibility to apoptosis in OCCCa Overexpression of LEFTY by TGF-β1/Smad signaling and/or other factors either activates the p53/p21^waf1^ pathway or induces p27^kip1^ expression, resulting in decreased cell proliferation. Its overexpression is also responsible for susceptibility to apoptosis due to the inhibition of the Smad2/XIAP axis and bcl2 expression, as well as the activation of the p53/bax pathway.

Together, our observations suggest a model for the functional role of LEFTY in OCCCa (Figure 9). Up-regulation of LEFTY expression by TGF-β1/Smad signaling and/or other factors results in decreased cell proliferation, probably due to either an activated p53/p21^waf1^ pathway or the induction of p27^kip1^ expression. Its overexpression is also associated with an enhancement of apoptotic features through the inhibition of Smad2-mediated *XIAP* transcription and decreased bcl2 expression, as well as activation of the p53/bax pathway. The pSmad2 may also participate in susceptibility to apoptosis in certain conditions. Thus, LEFTY may be an excellent OCCCa-specific molecular marker and has anti-tumor effects through modifying cell proliferation and susceptibility to apoptosis.

## MATERIALS AND METHODS

### Clinical cases

A total of 143 cases of OECa including 99 OCCCa, 13 OEmCa, 18 OSeCa, and 13 OMuCa, surgically resected at Kitasato University Hospital between 2000 and 2017, were selected from our patient records, according to 2014 criteria of the World Health Organization.[34] All tissues were routinely formalin-fixed (10%) and paraffin-embedded (FFPE). Of these, 16 FFPE samples (four of each subtype) were used for shotgun proteomics analysis. In addition, 42 fresh OECa samples (12 OCCCa, 11 OEmCa, 11 OMuCa, and eight OSeCa) were used for reverse transcription-polymerase chain reaction (RT-PCR) and/or western blot assays.

In OCCCa cases, none of the patients had received chemotherapy or any other preoperative treatment and most patients underwent postoperative adjuvant chemotherapy (administration of a paclitaxel, carboplatin, docetaxel, cisplatin-containing regimen). The mean age of patients was 55.3 years (range, 35 to 75 years). Forty-nine cases were subcategorized as clinical stage I, and 36 as stages II to IV, according to the criteria of the International Federation of Gynecology and Obstetrics (FIGO).[35] Ten were positive for nodal metastasis, while 75 were negative. Of 26 OCCCa patients with postoperative local tumor recurrence, six cases were available for further investigation of recurred tumors (Supplementary Table 1). Approval for this study was given by the Ethics Committee of the Kitasato University School of Medicine (B15-216).

### Shotgun proteomics analysis

Shotgun proteomics using FFPE samples was performed as described previously [36, 37]. Briefly, 15 μm FFPE sections were deparaffinized, rehydrated, and homogenized in protein extraction buffer (600 mM Tris-HCl pH8.8, 12 mM sodium lauryl sulfate, 12 mM sodium deoxycholate). Samples were incubated at 90°C for 2 h. After cooling to room temperature, reduced disulfides were alkylated by 55 mM iodoacetamide for 0.5 h and enzymatically digested overnight using trypsin (Promega, Madison, WI, USA) and lysyl endopeptidase (Wako Pure Chemical Industries Ltd, Osaka, Japan). The digested samples were acidified with 0.5% trifluoroacetic acid to precipitate surfactants and were desalted with C18-StageTips, followed by lyophilization [38]. The lyophilized samples were dissolved in 3% acetonitrile, and 0.1% formic acid. The peptides were injected into a trap column: C18 0.1×20 mm (Acclaim PepMap100; Thermo Fisher Scientific, Bremen, Germany), and an analytical column: C18 0.075×120mm (Nano HPLC Capillary Column; Nikkyo Technos, Tokyo, Japan), which was attached to an EASY-nLC 1000 liquid chromatograph (Thermo Fisher Scientific). The flow rate of the mobile phases was 300 nL/min, which consisted of (A) 0.1% formic acid, and (B) 0.1% formic acid and 90% acetonitrile. The mobile phase was programmed as follows: 0-8% B (0-2 min), 8%-32% B (2-132 min), 32-45% B (132-152 min), 45-100% B (152-153 min), and 100% B (153-165 min). Peptides separated by HPLC were introduced to the Q-Exactive mass spectrometer (Thermo Fisher Scientific).

The Q Exactive instrument was operated in a data-dependent mode to automatically switch between full scan MS and MS/MS acquisition. Full-scan MS spectra (m/z 350−1500) were acquired in the Orbitrap with a 70,000 resolution at m/z 200 after the accumulation of ions to a 3 × 10^6^ target value. The 10 most intense peaks with a charge state ≥2 from the full scan were selected with an isolation window of 2.0 Da and fragmented in the HCD collision cell with a normalized collision energy of 25%. Tandem mass spectra were acquired in the Orbitrap mass analyzer with a mass resolution of 17,500 at m/z 200 after accumulation of ions to a 2 × 10^5^ target value. The ion selection threshold was 1 × 10^5^ counts, and the maximum allowed ion accumulation times were 60 ms for full MS scans and 60 ms for tandem mass spectra. Typical mass spectrometric conditions were as follows: spray voltage, 2 kV; no sheath and auxiliary gas flow; heated capillary temperature, 250°C; and dynamic exclusion time, 60s.

Mass spectral data were processed, exported, and searched against a UniProt human database using SEQUEST by Proteome Discoverer (version 1.3; Thermo Fisher Scientific). Database search parameters were: peptide mass tolerance, 6 ppm; fragment tolerance, 15 ppm; enzyme was set to trypsin, allowing up to two missed cleavages; fixed modifications, carbamidomethyl (cysteine); and variable modifications, oxidation (methionine). The false discovery rate (FDR) was calculated by enabling the peptide sequence analysis using a decoy database. We used a 1% FDR as a cut-off to export results from the analysis. Quantitative analysis of shotgun proteomics data was achieved by spectral counting.

### Antibodies and reagents

Anti-LEFTY, anti-Smad2, and anti-phospho-Smad2 at serine 255 (pSmad2) antibodies were purchased from Abcam (Cambridge, MA, USA). Anti- XIAP, anti-bax, anti-β-catenin, anti-HNF-1β, and anti-p27^Kip1^ antibodies were bought from BD Biosciences (San Jose, CA, USA). Anti-p21^waf1^, anti-cyclin D1, anti-p53, anti-bcl2, and anti-Ki-67 antibodies were purchased from Dako (Copenhagen, Denmark). Anti-cyclin A, and anti-cleaved caspase 3 antibodies were from Novocastra (Newcastle, UK), and Cell Signaling Technology (Danvers, MA, USA), respectively. CDDP and the anti-β-actin antibody were purchased from Sigma-Aldrich Chemicals (St. Louis, MO, USA). Recombinant transforming growth factor (TGF)-β1 was purchased from R&D Systems (Minneapolis, MN, USA).

### Immunohistochemistry

IHC was performed using a combination of microwave-oven heating and polymer immunocomplex (Envision, Dako) methods, as described previously [39, 40].

For the evaluation of IHC findings, the scoring of nuclear and/or cytoplasmic immunoreactivity for LEFTY, pSmad2, and XIAP was performed, as described previously [39, 40]. Briefly, the proportion of immunopositive cells among the total number of counted cells was subdivided into five categories as follows: 0, all negative; 1, <10%; 2, 10-30%; 3, 30-50%; and 4, >50% positive cells. Immunointensity was also subclassified into four groups: 0, negative; 1, weak; 2, moderate; and 3, strong immunointensity. IHC scores were generated by the multiplication of values of the two parameters. In addition, cases were divided into high and low LEFTY categories on the basis of the mean±SD values of the LEFTY score. Nuclear immunopositivity for Ki-67 was also counted in at least 1000 cells in five randomly selected fields. LIs were calculated as the number per 100 cells, as described previously [39, 40].

### *In situ* hybridization

Riboprobes for LEFTY1 and LEFTY2 containing nucleotides 1022 to 1274 and 1186 to 1467 of the *LEFTY1* and *LEFTY2* genes (Table 2), respectively, were generated by *in vitro* transcription, and ISH assays were performed using the GenPoint Tyramide Signal Amplification System (Dako), as described previously [41]. The ISH signal score was determined on the basis of the percentage of ISH signal-positive cells (1, less than 10% positive cells; 2, 10-30%; 3, 30-50%; 4, more than 50%) and the ISH signal-intensity (0, none; 1, weak; 2, moderate; 3, strong) with the multiplication of values of the two parameters.

**Table 2:** Primer sequences used in this study

Gene	Assay		Sequence
LEFTY1	Promoter	Forward	5′-TGCTAGGCACCCTGTAGACA-3′
		Reverse	5′-AAGGCTGCAGGAGGGTCTCA-3′
	mRNA	Forward	5′-CTGCTGATGGACAAATGCTCTG-3′
	/ISH probe	Reverse	5′-ACTTTAGCCCAGATCCAGTGAC-3′
	Methylation	Forward	5′-TAGTTTTTAAGGTTTAGGGTGTG-3′
		Reverse	5′-TACTAACCCTACTCTTATCCC-3′
	sh 1207-1229	Forward	5′-GATCCCGACTTGTGTGTGTTTCTGAAGGCTTCC
			TGTCACCTTCAGAAACACACACAAGTCTTTTTTG-3′
		Reverse	5′-AATTCAAAAAAGACTTGTGTGTGTTTCTGAAGG
			TGACAGGAAGCCTTCAGAAACACACACAAGTCGG-3′
LEFTY2	Promoter	Forward	5′-TTCCCTGTTCTTCAAACACCGTCC-3′
		Reverse	5′-TCCTCTAGGGAGGTTGAAGGAGG-3′
	mRNA	Forward	5′-TCTAACTGAACGTGTGCATAG-3′
	/ISH probe	Reverse	5′-GAAGAATTTGCCAGAGAACAG-3′
XIAP	mRNA	Forward	5′-GCTAACTGATTGGAAGCCC-3′
		Reverse	5′-GTCCTTGAAACTGAACCCC-3′
	Promoter	Forward	5′-CTAGGTCAGCTTCTCGGTTCCAG-3′
		Reverse	5′-GAGAAACCCGGAGTCGTGAAACC-3′

ISH, *in situ* hybridization; sh, short-hairpin.

### Apoptosis and TdT-mediated dUTP-biotin nick end labeling assay

Apoptotic cells were identified in HE-stained sections, according to the criteria of Kerr et al [42]. A total of 20 fields were randomly selected, and the number of apoptotic cells was calculated by counting the mean number of apoptotic figures per 10 high power fields (HPFs), as described previously [39].

A TUNEL assay for the detection of apoptotic cells was also conducted using the *In Situ* Cell Death Detection Kit (Roche, Tokyo, Japan), according to the manufacturer's instructions. The number of positive cells was also analyzed by counting the mean number of TUNEL-positive cells per 10 HPFs.

### Plasmids and cell lines

Full-length cDNA for human LEFTY1, LEFTY2, and Smad2, which were from Open Biosystems (Huntsville, AL, USA), were subcloned into pcDNA3.1 (Invitrogen, Carlsbad, CA, USA). The human *LEFTY1* promoter (GeneBank accession number NM020997) between −3533 to +33 bp, the human *LEFTY2* promoter (NM003240) between −4805 to +42 bp, and the human *XIAP* promoter (NG007264) encompassing −432 to −67 bp (where +1 represents the transcription start site) were amplified by PCR and subcloned into the pGL-3B vector (Promega). The identity of all constructs was confirmed by sequencing prior to use. The sequences of PCR primers employed in this study are listed in Table 2.

TOV-21G and ES-2 cell lines were obtained from the American Type Culture Collection (Manassas, VA, USA), and the OVISE and Ishikawa cell lines were also from the National Institute of Biomedical Innovation (Osaka, Japan).

The LEFTY1 expression plasmid or empty vector was transfected into TOV-21G and ES-2 cells, and stable overexpressing clones were established as described previously [39, 40].

LEFTY1 specific shRNA oligonucleotides were designed using siDirect version 2 software. Single-stranded Lefty oligonucleotides were annealed and then cloned into *BamH*l-*EcoR*V sites of the RNAi-Ready pSIREN-RetroQ vector (Takara, Shiga, Japan), according to the manufacturer's instructions. The shLEFTY1 plasmid or non-specific shRNA constructs were transfected into OVISE cells, and stable knockdown clones were established.

### Transfection

Transfection was conducted using LipofectAMINE PLUS (Invitrogen), in duplicate or triplicate as described previously [38, 39]. Luciferase activity was assayed as described previously [39, 40].

### Reverse transcription- and real time-polymerase chain reaction

To start, cDNA was synthesized from 2 μg of total RNA. Amplification by conventional RT-PCR was carried out in the exponential phase to allow comparison among cDNAs synthesized from identical reactions using specific primers (Table 2). The intensity of individual signals was measured using ImageJ software version 1.41 (NIH, Bethesda, MD, USA). For quantitative analysis, real-time PCR was also conducted using a Power SYBR Green PCR Master Mix (Applied Biosystems, Foster City, CA, USA). Fluorescent signals were detected using the ABI 7500 real-time PCR System, and data were analyzed using the associated ABI 7500 System SDS Software (Applied Biosystems). Primers for the *GAPDH* gene were also applied as described previously [39, 40].

### Western blot assays

Cultured cell lines were lysed using RIPA buffer (50 mM/L, Tris-HCl [pH7.2], 1% Nonidet P-40, 0.5% sodium deoxycholate, 0.1% sodium dodecyl sulfate) and clinical samples were lysed directly with 2x Laemmli sample buffer (65 mM/L Tris-HCl [pH6.8], 5% 2-mercaptoethanol, 3% sodium dodecyl sulfate, 10% glycerol). Aliquots of the proteins were resolved by SDS-polyacrylamide gel electrophoresis, transferred to PVDF membranes, and probed with primary antibodies coupled with an ECL detection system (Amersham Pharmacia Biotechnology, Tokyo, Japan).

### Flow cytometry

Cells were fixed using 70% alcohol and stained with propidium iodide (Sigma-Aldrich) for analysis of apoptotic cells. The prepared cells were analyzed by flow cytometry using BD FACS Calibur flow cytometer (BD Biosciences) and CellQuest Pro software (BD Biosciences), as described previously [39, 40].

### Cell counting Kit-8 assay

The quantitation of viable cell number in proliferation and after CDDP treatment was carried out using a Cell Counting Kit-8 (CCK-8; Dojindo Lab, Kumamoto, Japan), according to the manufacturers’ instructions.

### Methylation analysis of *LEFTY1*

The methylation status of the *LEFTY1* was analyzed as described previously.[27] Briefly, genomic DNA extracted from cell lines using a Wizard Genomic DNA Purification kit (Promega) was treated by bisulfate using an EZ DNA Methylation-Gold kit (ZYMO Research, Orange, CA, USA). Bisulfate-treated DNA was amplified by PCR using specific primers as described previously. Amplicons were ligated into a pCR2.1 vector using the TA cloning kit (Invitrogen). Four positive clones were identified and sequenced from each sample. The DNA sequence generated was subjected to bioinformatics analysis to describe the CpG methylation status of these clones.

### Mutation analysis for *p53*

Exons 5 to 9 of the *p53* in TOV-21G and OVISE cells were amplified by PCR, and the products were subjected to direct sequencing PCR as described previously.[43]

### Statistics

Comparative data were analyzed using Kruskal-Wallis, Mann-Whitney *U*- and Chi-square tests, and Spearman's correlation coefficient, as appropriate. The cutoff for statistical significance was set as *p*<0.05.

## SUPPLEMENTARY MATERIALS FIGURES AND TABLES



## Abbreviations

LEFTY: left-right determination factor
OECa: ovarian epithelial carcinoma
OCCCa: ovarian clear cell carcinoma
OSeCa: ovarian serous carcinoma
OEmCa: ovarian endometrioid carcinoma
OMuCa: ovarian mucinous carcinoma
FFPE: formalin-fixed and paraffin-embedded
XIAP: X-linked inhibitor of apoptosis protein
TGF-β: transforming growth factor β
CDDP: cisplatin
IHC: immunohistochemistry
ISH: *in situ* hybridization
TUNEL: TdT-mediated dUTP-biotin nick end labeling
LI: labeling index
