# Supplementary material for: Identification of LEFTY as a molecular marker for ovarian clear cell carcinoma
Source: Oncotarget. 2017 Jun 29;8(38):63646–64. doi: 10.18632/oncotarget.18882 (PMC5609950; doi:10.18632/oncotarget.18882)
Supplement: Supplementary file 1 [file oncotarget-08-63646-s001.pdf]

Identification of LEFTY as a molecular marker for ovarian clear cell carcinoma

SUPPLEMENTARY MATERIALS

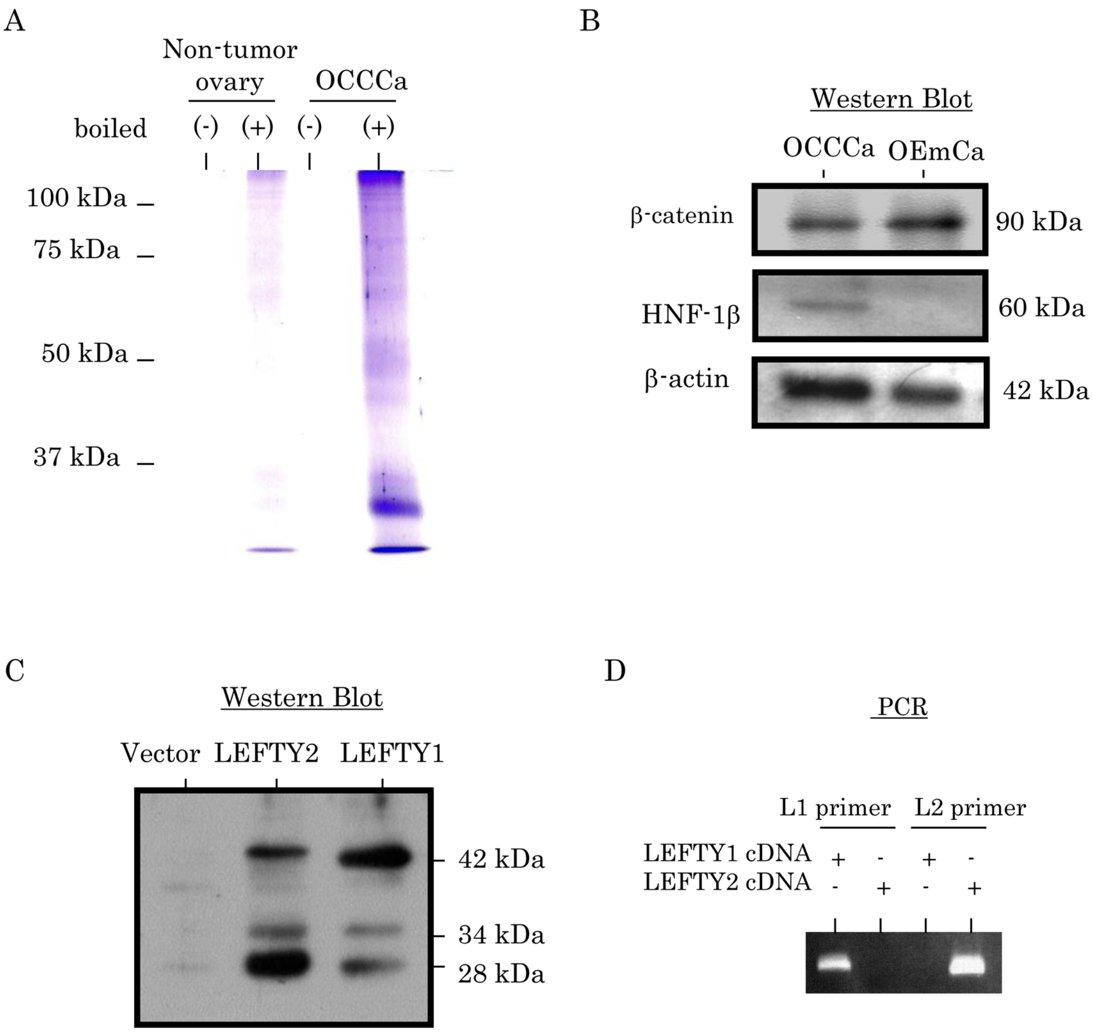

Supplementary Figure 1: (A) Coomassie Brilliant Blue staining of protein lysates extracted from formalin-fixed (10%) and paraffin-embedded (FFPE) samples using a high concentration Tris-buffer, with or without boiling. **(B)** Western blot analysis for the indicated proteins using protein lysates extracted from FFPE samples. **(C)** Western blot analysis of LEFTY protein expression using proteins extracted from LEFTY1- and LEFTY 2-transfected Ishikawa cells. **(D)** Conformation of specific primers for *LEFTY1* and *LEFTY2* when LEFTY1 and LEFTY2 cDNAs, respectively were transfected into Ishikawa cells.

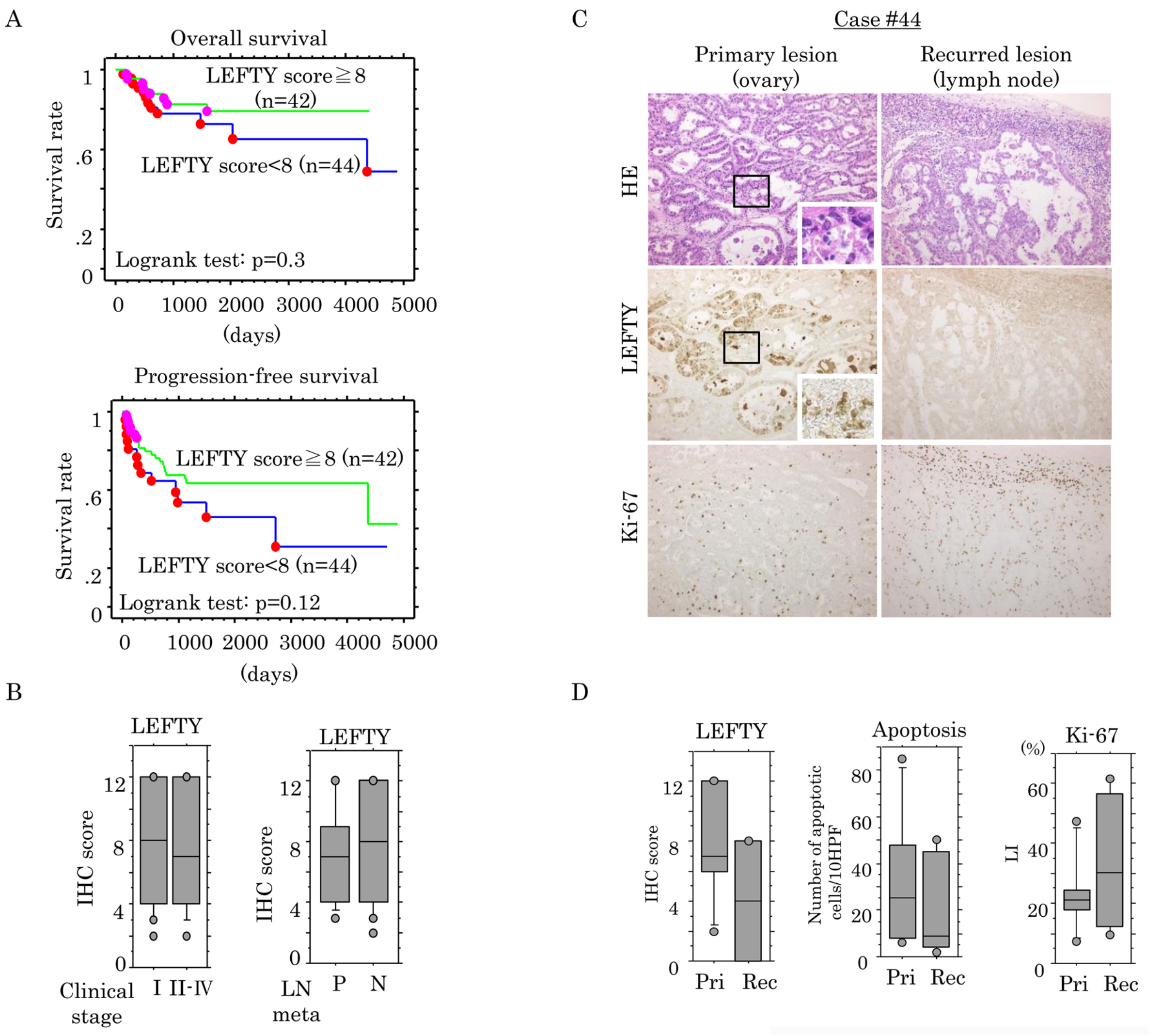

Supplementary Figure 2: (A) Relationship of LEFTY expression with overall survival and progression-free survival in OCCCas. **(B)** Relationship of LEFTY expression with clinical stage (left) and lymph node (LN) metastasis (meta; right). **(C)** Staining is by hematoxylin and eosin (HE) and by immunohistochemistry (IHC) for LEFTY and Ki-67 in semi-serial sections of OCCCa case #44 with postoperative tumor recurrence. Note the high LEFTY immunoreactivity in primary tumors, in contrast to a lack of immunostaining in recurred tumors within the lymph node. Original magnification, x200. **(D)** Relationship of LEFTY score, number of apoptotic cells, and Ki-67 labeling indices (LIs) between primary (Pri) and recurred (Rec) tumors in six OCCCa cases.

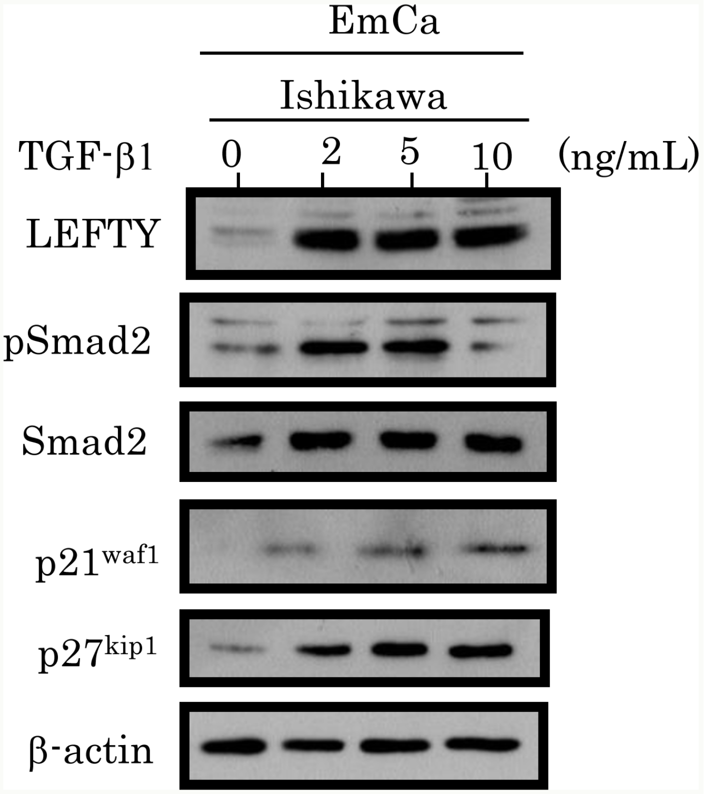

Supplementary Figure 3: Western blot analysis for the indicated proteins from Ishikawa cells after 2, 5, and 10 ng/mL TGF-β1 treatment for 24 hours.

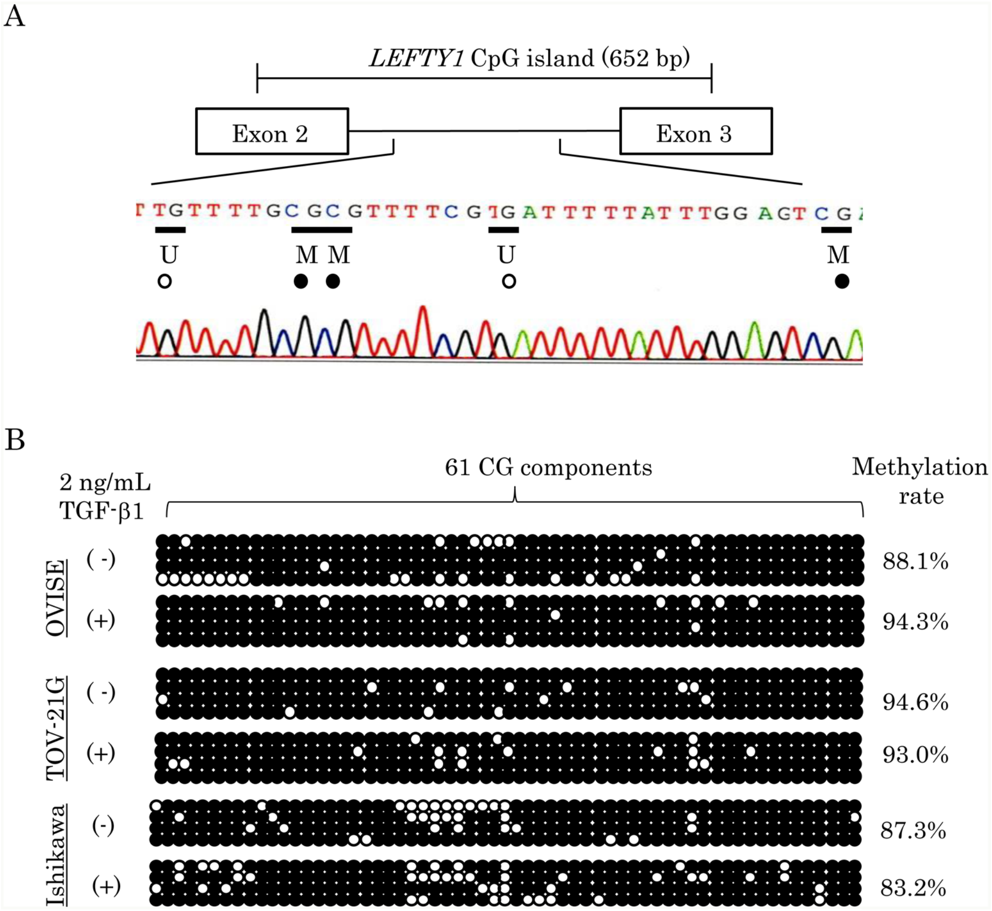

Supplementary Figure 4: Relationship between LEFTY1 methylation and TGF-β1. **(A)** CpG island DNA methylation analysis of the *LEFTY1* by sodium bisulfate sequencing. U, unmethylated (open circle); M, methylated (solid circle). **(B)** A total of 61 CpG islands were analyzed for DNA methylation. Each circle represents a CpG dinucleotide. Four clones are represented for each sequenced region before and after 2 ng/mL TGF-β1 treatment. Methylation percentages are indicated on the right. Open circle, unmethylated; solid circle, methylated.

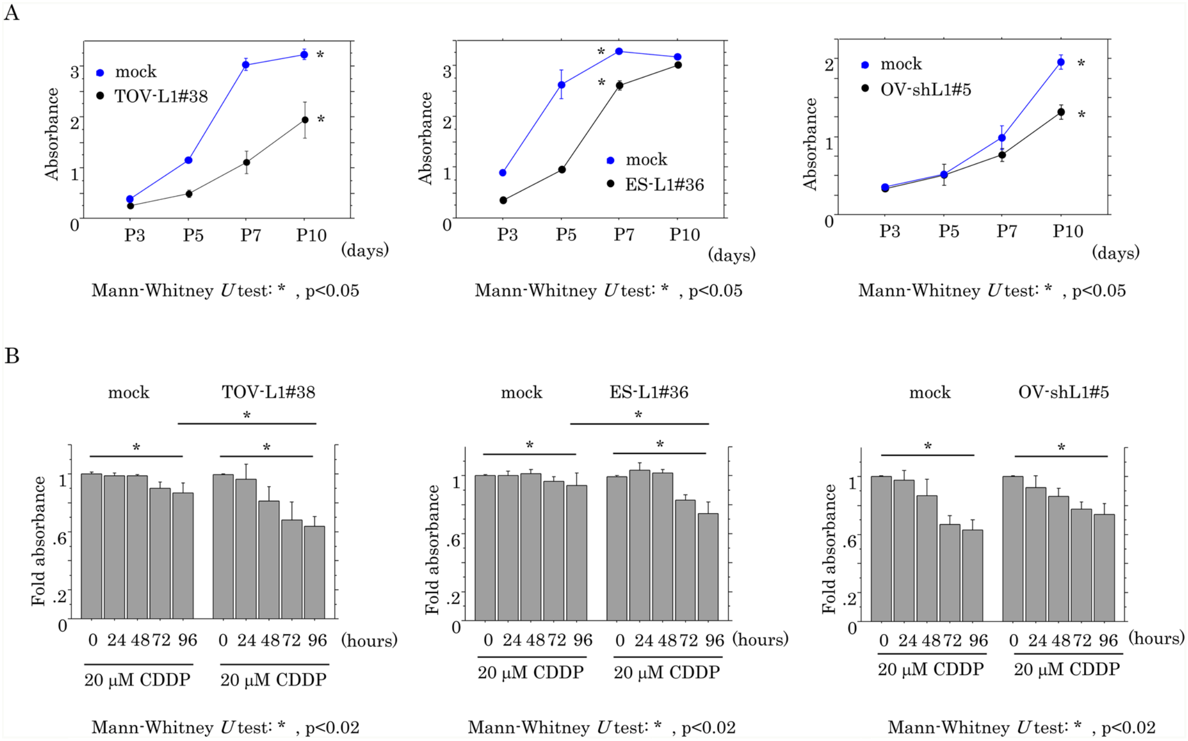

Supplementary Figure 5: CCK-8 assay for cell proliferation and after CDDP treatment. (A) TOV-L1#38 (left), ES-L1#36 (middle), and OV-shL1#5 (right) cells and their mock cells were seeded at $1x10^3$ cells in 96-wells. Viable cell numbers were quantitated by Cell Counting Kit-8 (CCK-8). Absorbance values are presented as mean±SD. P3, P5, P7, and P10 indicate 3, 5, 7, and 10 days after cell passage, respectively. This experiment was performed in triplicate using independent samples. **(B)** Treatment of TOV-L1#38 (left), ES-L1#36 (middle), and OV-shL1#5 (right) cells and their mock cells with 20 μM CDDP for the times shown. Cell viability was measured by CCK-8 kit. The absorbance in the absence of CDDP treatment (0 h) was set as 1 and relative absorbance values are presented as mean±SD. This experiment was performed in triplicate using independent samples.

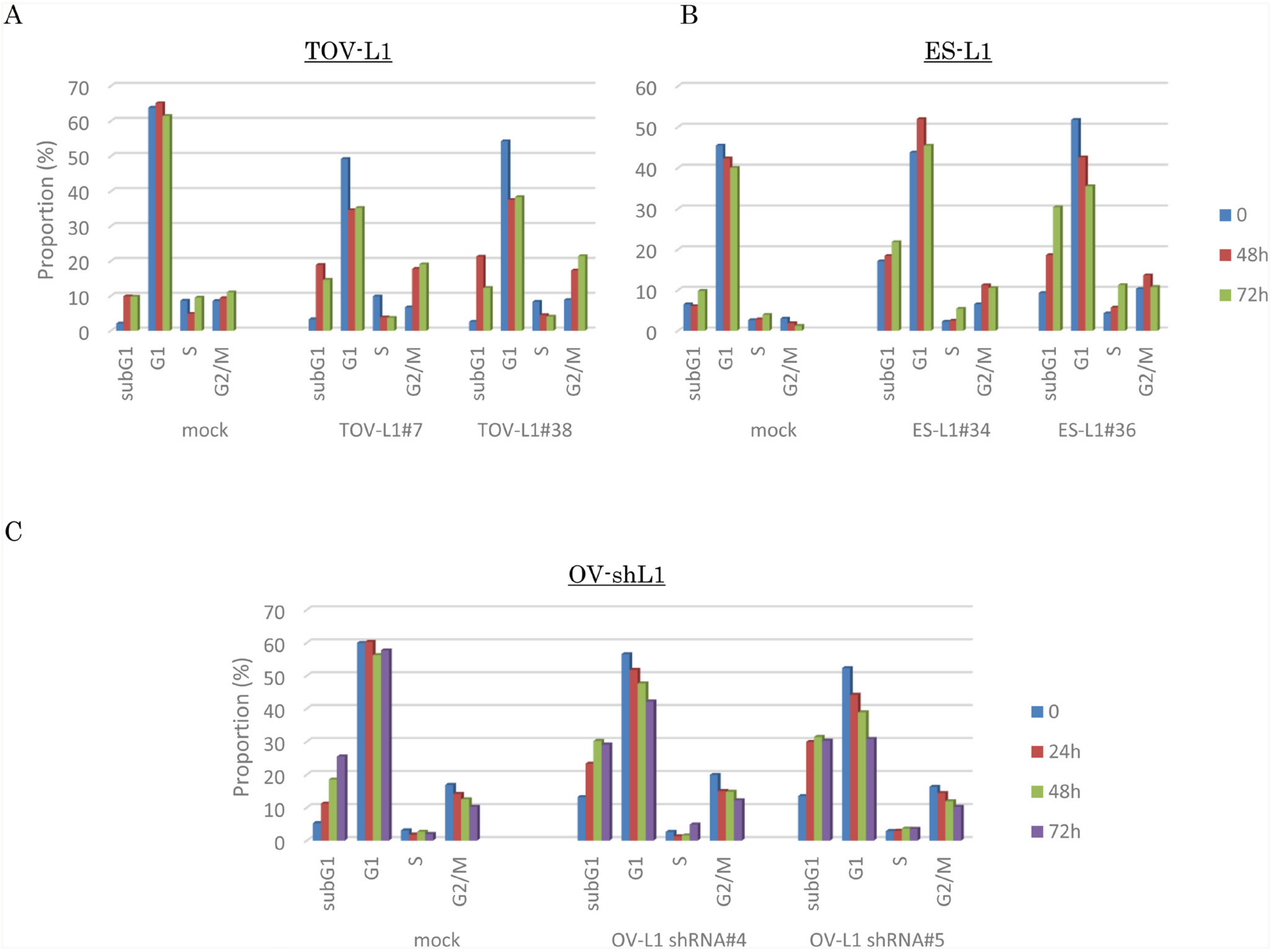

Supplementary Figure 6: After treatment with 20 µM CDDP, analysis of the percentage of subG1, G1, S, and G2/M fractions in TOV-L1 **(A)**, ES-L1 **(B)**, and OV-shL1 cells **(C)** for the times shown.

Supplementary Table 1: Summary of six OCCCa cases with local recurrence

Case no.	Age (years)	Clinical stage	Adjuvant chemotherapy	Primary tumor			Recurred tumor			
				LEFTY	Apoptosis	Ki-67 LI	Lesion	LEFTY	Apoptosis	Ki-67 LI
				(IHC score)	(10 H.P.F)	(%)		(IHC score)	(10 H.P.F)	(%)
#3	43	Ic	CPT+NEDA	12	85	7.3	peritoneum	6	45	28
#29	39	II	TC	8	12	18	peritoneum	8	6	9.4
#35	56	Ic	TC	6	48	24.2	lymph node	2	50	56.4
#42	37	II	DC, CPT+NEDA, CPT+CDDP, TC	6	12	18	peritoneum	8	12	12.3
#44	69	III	TC	8	12	24.4	lymph node	0	2	32.6
#45	56	Ic	TC, DC	2	37	47.5	lymph node	0	4	61.6

No., number; LI, labeling index; H.P.F, high power field; IHC, immunohistochemistry
CPT, irinotecan; NEDA, nedaplatin; TC, paclitaxel+carboplatin; DC, docetaxel+carboplatin; CDDP, cisplatin.

Supplementary Table 2: Summary of *in situ* hybridization scores for mRNA of LEFTY1 and LEFTY2 in 10 ovarian clear cell carcinoma cases

Case	*In situ* hybridization						IHC
	LEFTY1 mRNA (α -sense)			LEFTY2 mRNA (α -sense)			
	Positivity	Intensity	score	Positivity	Intensity	score	score
#1	3	4	12	2	3	6	12
#2	2	2	4	0	0	0	6
#3	1	4	4	1	2	2	6
#4	3	3	9	2	2	4	12
#5	3	3	9	0	0	0	12
#6	3	2	6	2	4	8	4
#7	2	3	6	1	1	1	12
#8	2	3	6	0	0	0	12
#9	2	1	2	0	0	0	2
#10	2	2	4	0	0	0	12

IHC, immunohistochemistry.

Supplementary Table 3: Summary of LEFTY1 and LEFTY2 protein profiles detected by shotgun proteomics in ovarian clear cell carcinomas

Protein	Peptide sequence	Spectral counts				Modification
		C1	C2	C3	C4	
LEFTY1	EVPTLDR	0	0	0	1	
LEFTY1	ADmEELVIPTHVR	2	2	0	1	M3(Oxidation)
LEFTY1	ADMEELVIPTHVR	2	4	0	2	
LEFTY1	AQYVALLQR	1	1	1	1	
LEFTY2	FLASEASTHLLVFGmEQR	0	2	0	1	M15(Oxidation)
LEFTY2	FLASEASTHLLVFGMEQR	0	9	0	4	
LEFTY1; LEFTY2	LPPNSELVQAVLR	2	5	1	4	
LEFTY1; LEFTY2	LFQEPVPK	1	1	1	1	
LEFTY1; LEFTY2	LFQEPVPKAALHR	0	1	0	0	
LEFTY1; LEFTY2	VTVEWLR	1	2	1	1	
LEFTY1; LEFTY2	TSLIDSR	1	1	0	1	
LEFTY1; LEFTY2	LVSVHESGWK	2	3	0	1	
LEFTY1; LEFTY2	AFDVTEAVNFWQQLSRPR	3	7	1	5	
LEFTY1; LEFTY2	QPLLLQVSVQR	1	5	0	2	
LEFTY1; LEFTY2	EHLGPLASGAHK	2	2	1	2	
LEFTY1; LEFTY2	EHLGPLASGAHKLVR	0	2	0	2	
LEFTY1	FASQGAPAGLGEPQLELHTL DLGDYGAQGDcDPEAPMTEGTR	0	1	0	0	C31(Carbamidomethyl)
LEFTY1; LEFTY2	QEmYIDLQGmK	0	1	0	0	M3(Oxidation); M10(Oxidation)
LEFTY1; LEFTY2	QEMYIDLQGmK	0	1	0	0	M10(Oxidation)
LEFTY1; LEFTY2	QEMYIDLQGMK	1	2	0	1	
LEFTY1	WAENWVLEPPGFLAYEcVGTcR	0	0	0	1	C17(Carbamidomethyl); C21(Carbamidomethyl)
LEFTY1	QPPEALAFK	1	2	0	1	
LEFTY1	QPPEALAFKWPFLGPR	0	1	0	0	
LEFTY1	WPFLGPR	4	5	1	4	
LEFTY1	QcIASETDSLPmIVSIK	0	1	0	0	C2(Carbamidomethyl); M12(Oxidation)
LEFTY1	QcIASETDSLPMIVSIK	0	3	0	1	C2(Carbamidomethyl)
LEFTY1; LEFTY2	TRPQVVSLPNmR	1	1	0	1	M11(Oxidation)
LEFTY1; LEFTY2	TRPQVVSLPNMR	2	3	1	2	
LEFTY1; LEFTY2	cScASDGALVPR	1	1	0	1	C1(Carbamidomethyl); C3(Carbamidomethyl)
Total number						
LEFTY1		28	58	8	36	
LEFTY2		18	49	6	29	
